# Vascular Endothelial Growth Factor Expression of Adipose-Derived Stromal Cells and Adipocytes Initiated from Fat Aspirations

**DOI:** 10.1007/s00266-024-04587-w

**Published:** 2024-12-10

**Authors:** Maryana Teufelsbauer, Sandra Stickler, Dennis C. Hammond, Gerhard Hamilton

**Affiliations:** 1 Clinics of Plastic and Reconstructive Surgery, Medical University of Vienna, 1090 Vienna, Austria; 2 Institute of Pharmacology, Medical University of Vienna, Waehringerstraße 13a, 1090 Vienna, Austria; 3 Center for Breast and Body Contouring, Grand Rapids, MI 49546 USA

**Keywords:** Lipofilling, Fat graft, Adipose-derived stromal cells, Vascularization, VEGF expression, Adipocytic differentiation

## Abstract

**Background:**

Fat grafting is frequently employed in aesthetic and reconstructive plastic surgery with a low complication rate. However, fat necrosis may occur in dependence of the mode of fat aspiration, processing of the tissue and graft size. Graft survival is critically dependent on the contained adipose-derived stromal cells (ADSCs), adipocyte precursors and their potential for vascular supply. This work investigated the potential role of the expression of vascular endothelial growth factor A (VEGF) and various cytokines by ADSCs and differentiated adipocytes as key factors of fat grafting.

**Methods:**

Adipokine expression of ADSCs and differentiated adipocytes were assessed using Proteome Profiler Arrays that detect 58 relevant proteins.

**Results:**

Collected fat grafts could be categorized according to their adipokine expression into VEGF^high^ and VEGF^low^ ADSCs groups, the former exhibiting higher content of VEGF-related angiopoietin-like 2, nidogen-1/entactin, CCL2/MCP-1 and elevated expression of IGFBPs in association with a fourfold higher VEGF expression. Differentiation of ADSCs into adipocytes increased VEGF concentrations in VEGF^low^ ADSCs but not in ADSCs exhibiting initial high VEGF concentrations. The adipocytes revealed high expression of HGF, leptin, CCL2/MCP-1, nidogen-1/entactin, M-CSF but lower induction of angiopoietin-like 2.

**Conclusion:**

Half of the ADSCs from fat grafts express high concentrations of VEGF and other adipokines that support angiogenesis and survival of this tissues following transfer. Differentiation of ADSC^low^ cells to adipocytes may make up for the initially low VEGF expression, but this activation is 7-10 days delayed compared to the VEGF^high^ ADSC cells and may fail to support angiogenesis from the beginning.

**No Level Assigned:**

This journal requires that authors assign a level of evidence to each submission to which Evidence-Based Medicine rankings are applicable. This excludes Review Articles, Book Reviews, and manuscripts that concern Basic Science, Animal Studies, Cadaver Studies, and Experimental Studies. For a full description of these Evidence-Based Medicine ratings, please refer to the Table of Contents or the online Instructions to Authors www.springer.com/00266.

## Introduction

Autologous fat grafting (AFG or lipofilling) is a common technique used in plastic and reconstructive surgery that involves the transfer of autologous fat tissue from one body site to another [1]. Niechajev and Śevćuk were the first to show that a low negative pressure and the use of cannulas with a diameter of 3, 4 or 6 mm decreased the number of damaged adipocytes [2]. Coleman also advised harvesting fat from donor sites by a 3-mm cannula at low negative pressure and grafting of fat in small portions to facilitate blood supply [3]. Coleman's technique increased survival of the fat grafts, making adoption of AFG more reliable. In aesthetic and reconstructive surgery, AFG/lipofilling is used for primary breast reconstruction especially after lumpectomies [4, 5]. Complications of fat grafting were low and consisted of cysts (0–20%), surgical-site infections (0–8%) and fat necrosis (0–58.4%) [1].

No significant differences in long-term retention between various techniques were found in AFG in the breast [6]. Fat graft degradation with reabsorption rates between 20 and 70% depending on the AFG protocol and length of follow-up constitutes a significant limitation. Fat grafts contain a high number of adipose-derived stromal cells (ADSCs) with multilineage potential that are key components of the adoption of the graft. However, clinical studies are discordant about the benefit of ADSC for AFG and fat grafting to the breasts [7]. For 170 cases of AFG for breast reconstruction, overall incidence of necrosis was 32.9 percent, with 47.8 percent in previously irradiated patients. Increased incidence of necrosis was associated with increasing fat graft volumes [8]. In fat necrosis, Kato et al. revealed that all adipocytes and ADSCs were dead, with the occupation of extracellular matrix, oil cysts, as well as calcification [9, 10]. Crucial steps for favorable results after AFG seem to be cautious manipulation of the adipocyte to minimize degradation of its fragile cell, washing of the lipoaspirate to eliminate debris and grafting of fat in vascularized sites [11]. The American Society of Plastic and Reconstructive Surgeons concluded that only 30% of injected fat can be expected to survive for 1-year [12].

The fat-grafting procedure involves aspiration of adipose tissue from a donor site, removal of cellular debris, acellular oil and infiltration fluid, followed by injection of the purified graft. Fat grafts have regenerative potential due to adult multipotent stromal cells in fat tissue, which has as many as 5000 cells/g of fat compared to 100–1000 stromal cells/ml of bone marrow [12, 13]. Actual ADSCs and preadipocytes might be the only cell types that survive grafting and replace adipocytes lost in the necrotic region. The stromal vascular fraction (SVF) of fat tissue is obtained from the dissociation of adipose tissue using enzymes and represent the simplest preparation of grafts [14]. SVF includes smooth muscle cells, fibroblasts, endothelial cells, pericytes, lymphocytes, macrophages and adipocytes progenitors in addition to ADSCs [15, 16]. Harvesting fat with a small diameter cannula has been made responsible for the damage of the sensitive adipocytes [17].

Although the mechanism of the regenerative potential of AFGs is not clear, it depends on an associated vasculogenic process [18]. Fat graft–induced neovascularization involves inflammatory responses, promotion of neo angiogenesis by adipokines and the differentiation of progenitor cells at the graft location. The contribution of the ADSCs is provided through soluble factors and cell-cell contacts that increase angiogenesis, formation of granulation tissue and re-epithelialization [19]. In particular, the integration of the graft tissue is driven by an efficient angiogenic effect, that is affected primarily by vascular endothelial growth factor A (VEGF) secretion and similar effectors [20]. Several days after AFG, revascularization of the graft is initiated from the periphery, but central fat regions vascularize only for small fragments [12]. Preadipocytes and ADSCs seem to comprise the single cells that survive AFG, and the variability of the characteristics of these cell types between individuals may be linked to the observed variability of the survival of fat grafts.

In the present study, we have collected several fat aspiration specimens, initiated cultures of ADSCs and assayed their expression of 58 adipokines to test for any predisposing and preexisting factors of AFG survival. Furthermore, three ADSC cultures were differentiated to adipocytes and subjected to the same adipokine determinations. According to these experiments, preexisting VEGF expression was characterized as an important factor likely to be essential for rapid vessel supply of AFGs.

## Methods

ADSCs were recovered from female patients following liposuction using 0.5 mm cannulas, with written consent of the patients according to Ethics Approval 366/2003 of the Ethics Committee of the Medical University of Vienna (Vienna, Austria). As described in a former publication, washed fat particles were incubated in RPMI-1640 medium (Sigma-Aldrich, St. Louis, MO, USA) supplemented with 30% fetal bovine serum (Eximus, Catus Biotech, Tutzing, Germany) and antibiotics (Sigma-Aldrich) until outgrowth of cells [21]. While ADSCs that attached to the 75 cm2 tissue culture flasks (Greiner Bio-One GmbH, Kremsmuenster, Austria) were further cultivated and expanded, residual fat tissue was discarded. Cells were kept under cell culture conditions (37°C, 5% CO2) and ADSCs were checked by flow cytometry for expression of CD73, CD90, and CD105 and negative reactivity for CD34 using a Cytomics FC500 fluorescence-activated cell sorting device (Beckman Coulter Germany GmbH, Krefeld, Germany). Conditioned cell culture supernatants were used for the analysis of the secreted adipokines.

Two fibroblast cell lines (FIB2 and FIB85), which were isolated in our laboratory from aneurysms, were used for controls. Several representative confluent ADSC cell cultures were differentiated into adipocytes using an adipocyte differentiation medium (Sigma-Aldrich). The Human Adipokine Array (Catalog #ARY024, R&D Systems, Minneapolis, Minneapolis) was used according to the manufacturer’s instructions to evaluate the levels of 58 adipokines.

### Statistical Analysis

Statistical significance was tested by *t* tests, and a value of *p* < 0.05 was regarded as a significant difference. The reference spots provided in the Human Adipokine Arrays were used to calibrate individual chemiluminescence intensities, while pixel values were normalized for comparability. Experiments were performed in duplicate and arrays evaluated using Quickspot (Ideal Eyes System, Bountiful, UT, USA) and Origin 9.1 software (OriginLab, Northampton, MA, USA).

## Results

**Fig. 1:**
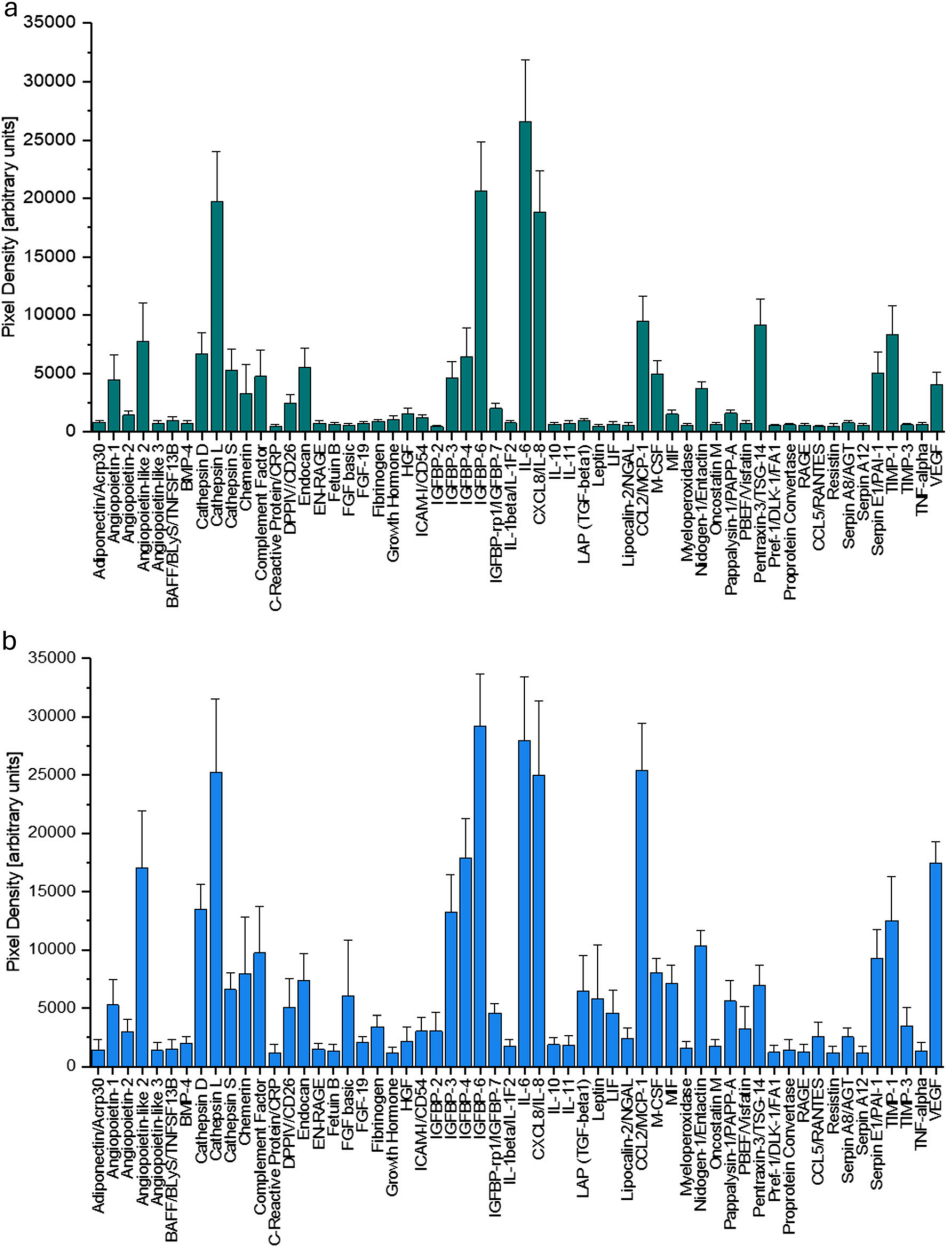
These graphs depict the levels of 39 obesity-related molecules that accompany **a** a low VEGF (vascular endothelial growth factor A) and **b** a high VEGF expression. These data were evaluated via a Human Adipokine Array (Catalog #ARY024, R&D Systems, Minneapolis, Minneapolis). Abbreviations are as follows: TNF superfamily member 13b (BAFF/BlyS/TNFSF13B), bone morphogenetic protein 4 (BMP4), complement factor 9 (complement factor), dipeptidyl peptidase 4 (DPPIV/CD26), S100 calcium binding protein A12 (EN-RAGE), fibroblast growth factor 2 (FGF basic), fibroblast growth factor 19 (FGF-19), hepatocyte growth factor (HGF), intercellular adhesion molecule 1 (ICAM-I/CD54), insulin-like growth factor binding protein 2 (IGFBP-2), insulin-like growth factor binding protein 3 (IGFBP-3), insulin-like growth factor binding protein 4 (IGFBP-4), insulin-like growth factor binding protein 6 (IGFBP-6), insulin-like growth factor binding protein 7 (IGFBP-rp1/IGFBP-7), interleukin 1 Beta (IL-1beta/IL-1F2), interleukin 6 (IL-6), interleukin 8 (8CXCL8/IL-8), interleukin 10 (IL-10), interleukin 11 (IL-11), laryngeal adductor paralysis (LAP), transforming growth factor beta 1 (TGF-beta1), LIF interleukin 6 family cytokine (LIF), C-C motif chemokine ligand 2 (CCL2/MCP-1), colony-stimulating factor 1 (M-CSF), macrophage migration inhibitory factor (MIF), delta-like non-canonical notch ligand 1 (Pref-1/DLK-1/FA1), advanced glycosylation end-product specific receptor (RAGE), C-C motif chemokine ligand 5 (CCL5/RANTES), TIMP metallopeptidase inhibitor 1 (TIMP-1), TIMP metallopeptidase inhibitor 3 (TIMP-3), tumor necrosis factor (TNF-alpha)

Between ADSCs revealing high (*n*=5) and low (*n*=6) VEGF levels, several other significant differences between adipokines could be found. angiopoietin-like 2, cathepsin D and L, intercellular adhesion molecule 1 (ICAM-I/CD54), insulin-like growth factor binding proteins (IGFBP) 2, 4 and 7, as well as IL-8, C-C Motif Chemokine Ligand 2 (CCL2/MCP-1), M-CSF and Serpin E1/PAI-1 were significantly higher expressed in VEGF^high^ ADSCs compared to the significantly lower expression in the VEGF^low^ specimens (Fig. 1a, b).

**Fig. 2:**
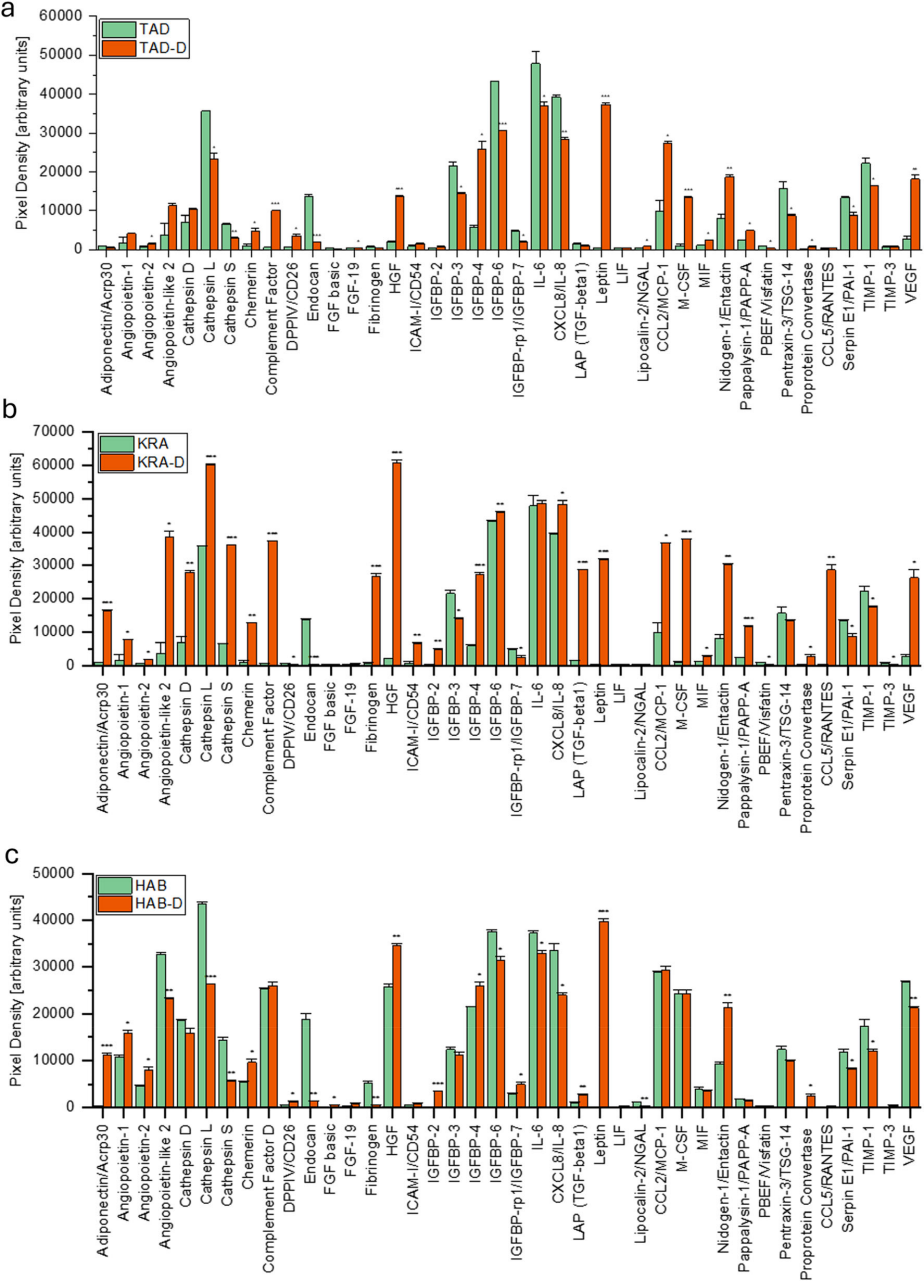
These graphs depict the levels of 40 obesity-related molecules before and after differentiation into adipocytes. These data were calculated via two independent Human Adipokine Arrays each (Catalog #ARY024, R&D Systems, Minneapolis, Minneapolis) on adipose-derived stromal cells and those differentiated into adipocytes using an adipocyte differentiation medium (-D). **a** TAD and TAD-D cells, **b** KRA and KRA-D cells and **c** HAB and HAB-D cells. Abbreviations are as follows: TNF superfamily member 13b (BAFF/BlyS/TNFSF13B), bone morphogenetic protein 4 (BMP4), complement factor 9 (complement factor), dipeptidyl peptidase 4 (DPPIV/CD26), S100 calcium binding protein A12 (EN-RAGE), fibroblast growth factor 2 (FGF basic), fibroblast growth factor 19 (FGF-19), hepatocyte growth factor (HGF), intercellular adhesion molecule 1 (ICAM-I/CD54), insulin-like growth factor binding protein 2 (IGFBP-2), insulin-like growth factor binding protein 3 (IGFBP-3), insulin-like growth factor binding protein 4 (IGFBP-4), insulin-like growth factor binding protein 6 (IGFBP-6), insulin-like growth factor binding protein 7 (IGFBP-rp1/IGFBP-7), interleukin 1 Beta (IL-1beta/IL-1F2), interleukin 6 (IL-6), interleukin 8 (8CXCL8/IL-8), interleukin 10 (IL-10), interleukin 11 (IL-11), laryngeal adductor paralysis (LAP), transforming growth factor beta 1 (TGF-beta1), LIF interleukin 6 family cytokine (LIF), C-C motif chemokine ligand 2 (CCL2/MCP-1), colony-stimulating factor 1 (M-CSF), macrophage migration inhibitory factor (MIF), delta-like non-canonical notch ligand 1 (Pref-1/DLK-1/FA1), advanced glycosylation end-product specific receptor (RAGE), C-C motif chemokine ligand 5 (CCL5/RANTES), TIMP metallopeptidase inhibitor 1 (TIMP-1), TIMP metallopeptidase inhibitor 3 (TIMP-3), tumor necrosis factor (TNF-alpha), VEGF (vascular endothelial growth factor A)

Two VEGF^high^ and one VEGF^low^ ADSC lines were differentiated into adipocytes as previously demonstrated. A comparison of ADSC line TAD and TAD-D, where cells were differentiated into adipocytes, showed increased levels of VEGF, angiopoietin-2, Chemerin, complement factor 9, hepatocyte growth factor (HGF), leptin, Lipocalin-2/NGAL, CCL2/MCP-1, M-CSF, macrophage migration inhibitory factor (MIF) and nidogen-1/entactin. In contrast, cathepsins L and S, endocan, FGF-19, IGFBP-3, 6, 7, as well as IL-6 and IL-8, along with protease and inhibitors decreased after differentiation of TAD ADSCs into adipocytes (Fig. 2a).

Another comparison of the ADSC line KRA and its differentiated adipocytes (KRA-D) showed significant increases in VEGF, angiopoietin-2 and angiopoietin-like 2, cathepsin D, L and S, HGF, IGFBP-2, 4 and 6, IL-8, leptin, CCL2/MCP-1, M-CSF, nidogen-1/entactin and C-C Motif Chemokine Ligand 5 (CCL5/RANTES). In contrast, adiponectin/Acrp3, angiopoietin-1, IGFBP-3 and 7, as well as E1/PAI-1, TIMP-1 and 3, were significantly decreased after adipocytic differentiation (Fig. 2b).

In the ADSC line HAB, where VEGF was already high before differentiation, there is a slight decrease in its amount after differentiation to HAB-D. Adiponectin/Acrp30, angiopoietin-1 and FGF basic, HGF, IGFBP-2, 4 and 7, LAP (TGF-beta1), leptin, nidogen-1/entactin, TIMP-1 increased significantly after differentiation. In contrast, angiopoietin-like 2, cathepsin L and S, endocan, IGFBP-6, IL-6 and IL-8 as well as Serpin E1/PAI-1 showed significant decreases in expression (Fig. 2c).

**Fig. 3:**
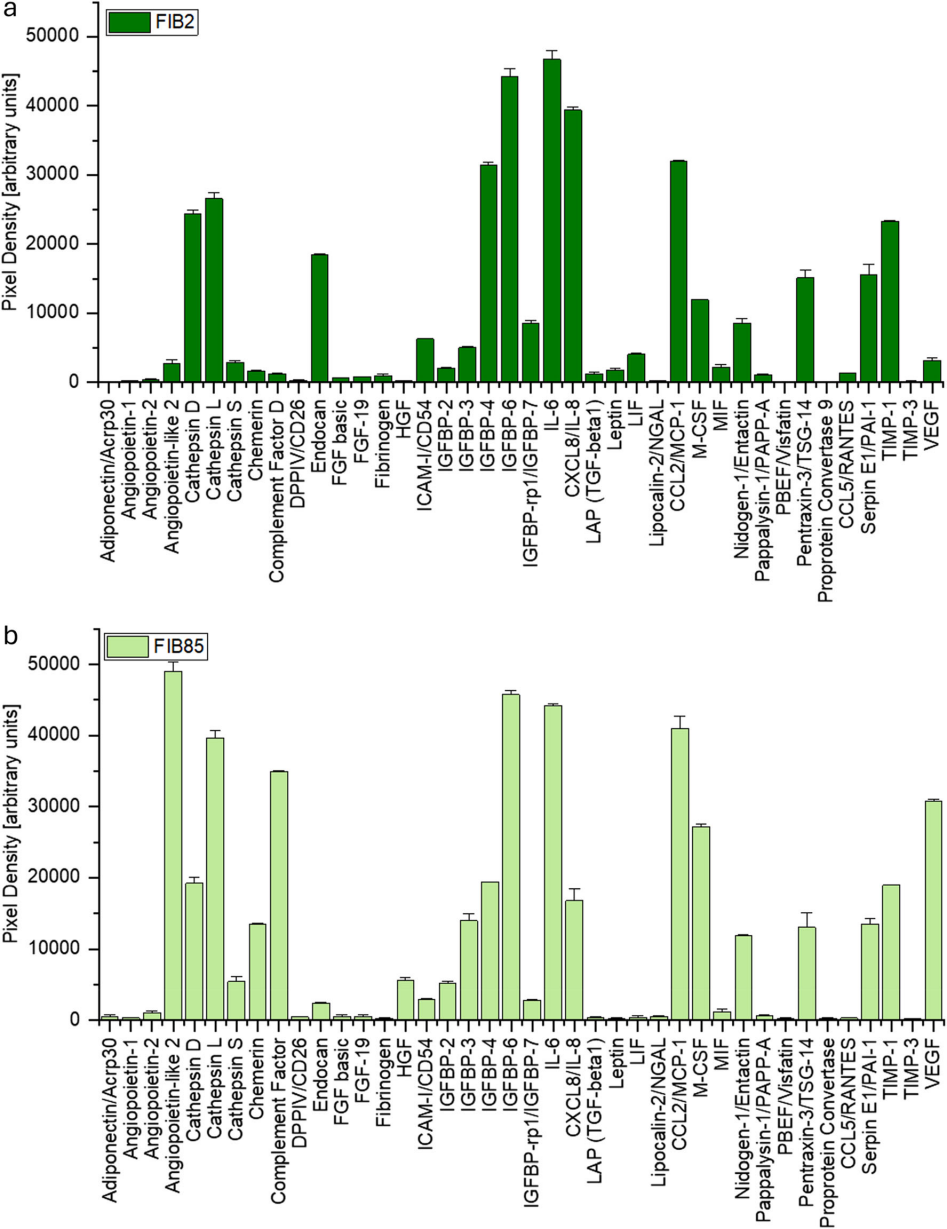
The levels of 40 adipokines of fibroblast line **a** FIB2 and **b** FIB85 are depicted in these graphs. These data were calculated via two independent Human Adipokine Arrays each (Catalog #ARY024, R&D Systems, Minneapolis, Minneapolis). Abbreviations are as follows: TNF superfamily member 13b (BAFF/BlyS/TNFSF13B), bone morphogenetic protein 4 (BMP4), complement factor 9 (complement factor), dipeptidyl peptidase 4 (DPPIV/CD26), S100 calcium binding protein A12 (EN-RAGE), fibroblast growth factor 2 (FGF basic), fibroblast growth factor 19 (FGF-19), hepatocyte growth factor (HGF), intercellular adhesion molecule 1 (ICAM-I/CD54), insulin-like growth factor binding protein 2 (IGFBP-2), insulin-like growth factor binding protein 3 (IGFBP-3), insulin-like growth factor binding protein 4 (IGFBP-4), insulin-like growth factor binding protein 6 (IGFBP-6), insulin-like growth factor binding protein 7 (IGFBP-rp1/IGFBP-7), interleukin 1 Beta (IL-1beta/IL-1F2), interleukin 6 (IL-6), interleukin 8 (8CXCL8/IL-8), interleukin 10 (IL-10), interleukin 11 (IL-11), laryngeal adductor paralysis (LAP), transforming growth factor beta 1 (TGF-beta1), LIF interleukin 6 family cytokine (LIF), C-C motif chemokine ligand 2 (CCL2/MCP-1), colony-stimulating factor 1 (M-CSF), macrophage migration inhibitory factor (MIF), delta-like non-canonical notch ligand 1 (Pref-1/DLK-1/FA1), advanced glycosylation end-product specific receptor (RAGE), C-C motif chemokine ligand 5 (CCL5/RANTES), TIMP metallopeptidase inhibitor 1 (TIMP-1), TIMP metallopeptidase inhibitor 3 (TIMP-3), tumor necrosis factor (TNF-alpha), VEGF (vascular endothelial growth factor A)

The fibroblast lines FIB2 and FIB85 showed similar levels of adipokines in comparison with ADSCs. Both fibroblast lines showed significant differences between each other regarding, e.g., VEGF (870% difference in VEGF), angiopoietin-like 2, cathepsin D, L and S, IGFBP-2, 3, 4 and 7, CCL2/MCP-1 and nidogen-1/entactin. Both fibroblast cell lines lack expression of leptin and FIB2 VEGF^low^ additionally exhibits low levels of angiopoietin-like (Fig. 3a, b).

## Discussion

Adipose tissue is present in large quantities and represents a suitable filler for remodeling purposes in plastic surgery. Major complications were noted in 3.2% of an AFG cohort vs 2.3% in the corresponding breast implant cohort. However, lack of documentation and controlled trials hamper the assessment of the complication rates of AFG [22]. Open critical issues for AFG include the occurrence of oil cysts or fat necrosis, mammographic abnormalities and the most effective technique to achieve a high fat graft survival. Fat resorption rates of 20–70% within one year have been reported, in particular for large-volume AFG, but numerous differences in the detailed procedures impede comparisons between the studies [23, 24]. In an innovative breast surgical hybrid method, relatively small fat implants can be enlarged by AFGs, especially to optimize the contours [25].

Fat is composed of adipocytes and other cells of the stromal vascular fraction (SVF), including immune cells (e.g., macrophages and lymphocytes), endothelial cells and their progenitors, smooth muscle cells, pericytes and ADSCs that could be released by collagenase treatment and used in assisted AFG [26, 27]. Though AFGs are successfully used in plastic surgery, little is known about the regulation of the viability of adipocytes and the development of preadipocytes. ADSCs constitute the cellular key factor for fat graft survival because of their ability to differentiate and secrete growth factors [28, 29]. Tests of cell viability have demonstrated that adipocytes are very fragile and have a poor tolerance to mechanical stress and ischemia [10, 30]. Preadipocytes are more resistant to hypoxic and traumatic damage during harvesting, processing and replanting because of a low metabolic activity and a marginal oxygen consumption rate compared to mature adipocytes [12]. It is supposed that the major contributor of effective fat tissue transplantation is the survival of ADSCs in the stromal cell fraction and their differentiation potential to regenerate adipocytes [3].

ADSCs exert their positive effects on fat graft survival and tissue repair mainly through the release of adipokines [31]. For example, leptin administration to fat transplants promoted angiogenesis and reduced the resorption rate of the lipoaspirates [32]. Re-establishment of the blood supply starts from day 7 after implantation and institutes functional vessel density 10–15 days after transplantation. ADSCs stimulate neovascularization through growth factors/adipokines, including VEGF and insulin-like growth factor 1 (IGF-1) [33]. Other mediators produced comprise factors such as leptin, VEGF, HGF, FGF basic, TGF-beta1, IL-8, platelet-derived growth factor (PDGF), PlGF (Phosphatidylinositol Glycan Anchor Biosynthesis Class F), or C-X-C motif chemokine ligand 12 (SDF-1) that are involved in angiogenesis [34–36]. In turn, FGFs and VEGFs are promoters for ADSC proliferation, migration, attachment, and endothelial differentiation [37]. Accordingly, fat grafts supplemented with ADSCs exhibit a higher capillary density [38, 39]. In a similar way, fat grafts enriched in proangiogenic factors improved the graft viability through increased vascularization [40]. Furthermore, ADSCs have immunomodulatory effects diverting the polarization of macrophages toward a reparative phenotype [18, 41]. In addition, CCL2/MCP-1 is a cytokine that also recruits macrophages, facilitates macrophage repolarization and results in accelerated tissue repair. Our arrays identified ADSC-derived nidogen-1 that constitutes an important basement membrane component, whose interactions in particular with laminin, collagen IV and perlecan contributes to tissue repair caused ischemic conditions during grafting [42, 43]. Nidogen-1 and −2 are found in 3T3-L1 preadipocytes, adipose tissue, adipocytes and bone marrow stromal cells, maintaining adipocyte basal lamina stability in combination with type IV collagen and laminins [44]. The strong upregulation of nidogen-1 during initial differentiation of 3T3-L1 preadipocytes demonstrates its key role for adipose tissue morphogenesis.

Our adipokine assays yielded the expected distribution of these reported mediators in ADSCs, showing expression of VEGF, angiopoietin-2 like, CCL2/MCP-1, nidogen-1, FGF, IL-6, IL-8 and various cathepsins and IGF-1 binding proteins. Surprisingly, the 11 donor fat aspirates differed markedly in their VEGF expression, exhibiting approximately fourfold higher levels in the 5 VEGF^high^ specimens compared to the VEGF^low^ fraction. Angiopoietin-like 2, intercellular adhesion molecule 1 (ICAM-I/CD54), insulin-like growth factor binding proteins (IGFBP) 2, 4 and 7, as well as IL-8, C-C Motif Chemokine Ligand 2 (CCL2/MCP-1) and M-CSF have proangiogenic characteristics in conjunction with elevated VEGF. Between ADSCs lines revealing high (*n*=5) and low (*n*=6) VEGF levels (difference of 334% in VEGF), several other significant differences between adipokines could be found. Angiopoietin-like 2, cathepsins D and L, ICAM-I/CD54, IGFBP-2, 4 and 7, as well as IL-8, C-C motif Chemokine Ligand 2 (CCL2/MCP-1), M-CSF and Serpin E1/PAI-1 were significantly higher expressed in VEGF^high^ ADSCs compared to the lower expression in the VEGF^low^ specimens (Fig. 1a, b).

The ADSCs possess multilineage differentiation potential and can be easily transformed into adipocytes. The typical adipocyte differentiation medium is supplemented with serum, 3-isobutyl-1-methylxanthine, indomethacin, dexamethasone, and insulin [45]. Lipid droplets start to develop after about one week, with their number increasing over time until after 12–14 days of treatment mature adipocytes are visible [46]. Two VEGF^high^ and one VEGF^low^ ADSC line were differentiated into adipocytes as previously demonstrated [20]. A comparison of the adipokine expression of the ADSC line TAD and the corresponding differentiated TAD-D adipocytes showed increased levels of VEGF, angiopoietin-like 2, Chemerin, complement factor, HGF, leptin, Lipocalin-2/NGAL, CCL2/MCP-1, M-CSF, MIF and nidogen-1/entactin in the adipocytes (Fig. 2a). In contrast, cathepsins L and S, endocan, FGF-19, IGFBP-3, 6 and 7, as well as IL-6 and protease and inhibitors decreased after differentiation of TAD ADSCs into adipocytes (Fig. 2a). Another comparison of the ADSC line KRA and its differentiated adipocytes (KRA-D) showed significant increases in VEGF, angiopoietin-2 and angiopoietin-like 2, cathepsins D, L and S, HGF, IGFBP-2, 4 and 6, IL-8, leptin, CCL2/MCP-1, M-CSF, nidogen-1/entactin and C-C Motif Chemokine Ligand 5 (CCL5/RANTES). In contrast, adiponectin/Acrp3, angiopoietin-1, IGFBP-3 and 7 and Serpin E1/PAI-1, TIMP-1 and 3, were significantly decreased after adipocytic differentiation (Fig. 2b). Although adipocytic differentiation increased the low-level VEGF concentrations, this would be significantly delayed in time compared to the VEGF^high^ fat aspirates.

In the ADSC line HAB, where VEGF was already high before differentiation into adipocytes, there was a slight decrease in this important mediator after differentiation to HAB-D. Adiponectin/Acrp30, angiopoietin-1 and FGF basic, HGF, IGFBP-2, 4 and 7, LAP (TGF-beta1), leptin, nidogen-1/entactin, TIMP-1 increased significantly after differentiation. In contrast, angiopoietin-like 2, cathepsin L and S, endocan, IGFBP-6, IL-6 and IL-8 as well as Serpin E1/PAI-1 showed significant decreases in their expression (Fig. 2c). Thus, in the VEGF^high^ HAB, adipocytic differentiation did not further increase VEGF and showed reduced angiopoietin-like 2, indicating less angiogenic activity. Fibroblasts can be differentiated into adipocytes [47]. The fibroblast lines FIB2 and FIB85 showed similar levels of adipokines in comparison with ADSCs. Both fibroblast lines showed significant differences between each other regarding, e.g., VEGF (870% difference in VEGF) and other adipokines (Fig. 3a, b). Both fibroblast cell lines lack expression of leptin and FIB2 VEGF^low^ additionally exhibits low levels of angiopoietin-like 2.

In conclusion, ADSCs derived from fat aspirations may be categorized into VEGF^high^ and VEGF^low^ populations that may predict different fates after grafting owed to the immediate support of angiogenesis due to the presence of high VEGF concentrations. Adipocytic differentiation of VEGF^low^ ADSCs can increase their VEGF levels similar to VEGF^high^ ADSCs, but this process is delayed during differentiation and will not support the survival of grafts in the initial phase after grafting. Thus, donor-to-donor variation could also be imputed to ADSCs, initial tissue inflammatory state or tissue origin as proposed before [28].

## Abbreviations

ADSCs: Adipose-derived stromal cells
AFG: Autologous fat grafting
BAFF/BlyS/TNFSF13B: TNF superfamily member 13b
BMP4: Bone Morphogenetic Protein 4
CCL2/MCP-1: C-C motif chemokine ligand 2
CCL5/RANTES: C-C motif chemokine ligand 5
Complement factor: Complement factor 9
DPPIV/CD26: Dipeptidyl peptidase 4
EN-RAGE: S100 calcium binding protein A12
FGF: Fibroblast growth factor
FGF basic: Fibroblast growth factor 2
FGF-19: Fibroblast growth factor 19
HGF: Hepatocyte growth factor
ICAM-I/CD54: Intercellular adhesion molecule 1
IGF-1: Insulin-like growth factor 1
IGFBP-2: Insulin-like growth factor binding protein 2
IGFBP-3: Insulin-like growth factor binding protein 3
IGFBP-4: Insulin-like growth factor binding protein 4
IGFBP-6: Insulin-like growth factor binding protein 6
IGFBP-rp1/IGFBP-7: Insulin-like growth factor binding protein 7
IL-1beta/IL-1F2: Interleukin 1 Beta
IL-6: Interleukin 6
8CXCL8/IL-8: Interleukin 8
IL-10: Interleukin 10
IL-11: Interleukin 11
MIF: Macrophage migration inhibitory factor
M-CSF: Colony-stimulating factor 1
LAP: Laryngeal adductor paralysis
LIF: Interleukin 6 family cytokine
PDGF: Platelet-derived growth factor
PlGF: Phosphatidylinositol glycan anchor biosynthesis class F
Pref-1/DLK-1/FA1: Delta-like non-canonical notch ligand 1
RAGE: Advanced glycosylation end-product specific receptor
SDF-1: C-X-C motif chemokine ligand 12
SVF: Stromal vascular fraction
TGF-beta1: Transforming growth factor beta 1
TIMP-1: TIMP metallopeptidase inhibitor 1
TIMP-3: TIMP metallopeptidase inhibitor 3
TNF-alpha: Tumor necrosis factor
VEGF: Vascular endothelial growth factor A
